# Two cases of white globe appearance in non-cancerous stomach

**DOI:** 10.3332/ecancer.2018.856

**Published:** 2018-08-07

**Authors:** Masaya Iwamuro, Takehiro Tanaka, Hiroyuki Sakae, Yasushi Yamasaki, Hiromitsu Kanzaki, Seiji Kawano, Yoshiro Kawahara, Hiroyuki Okada

**Affiliations:** 1 Department of Gastroenterology and Hepatology, Okayama University Graduate School of Medicine, Dentistry, and Pharmaceutical Sciences, 2-5-1 Shikata-cho, Kita-ku, Okayama 700-8558, Japan; 2 Department of Pathology, Okayama University Hospital, Okayama 700-8558, Japan; 3 Department of Endoscopy, Okayama University Hospital, Okayama 700-8558, Japan

**Keywords:** white globe appearance, intraglandular necrotic debris, gastric cancer, acid secretion inhibitor

## Abstract

In this report, we describe two patients with white globe appearance in the non-cancerous stomach. The patient in Case 1 was an 82-year-old Japanese man who had been taking vonoprazan, dimethicone, acotiamide, sitagliptin, candesartan, dutasteride, etizolam and zolpidem. The patient in Case 2 was a 74-year-old Japanese woman who had been taking esomeprazole, rebamipide, sitagliptin, candesartan, ezetimibe, mirabegron, levocetirizine, zolpidem and lactobacillus preparation. In both cases, endoscopy revealed multiple white spots in the stomach. Magnifying endoscopy and blue laser imaging revealed a slightly elevated, round, white substance. Biopsied specimens from the lesions contained parietal cell protrusions and fundic gland cysts. Intraglandular necrotic debris was absent. Consequently, microscopic features in these cases were different from those reported previously for white globe appearance observed in gastric cancer lesions. These results indicate that white globe appearance can be observed in non-cancerous stomach. Although the macroscopic features could be confusing or misleading, thorough endoscopic observation and pathological analysis of white globe appearance will aid oncologists and endoscopists in differentiating between cancer-related lesions and non-cancerous lesions.

## Introduction

White globe appearance was first reported in 2015 by Doyama *et al* [1] as a small, white lesion in the gastric mucosa. Doyama *et al* suggested that the finding pathologically corresponded to a dilated gastric gland containing eosinophilic material with necrotic epithelial fragments, called intraglandular necrotic debris [1, 2]. It is noteworthy that the white globe appearance is found within the margin of the cancerous gastric epithelium.

The presence of white globe appearance in the stomach has been reported to be highly indicative of cancer [1]. However, it can also be observed in non-cancerous gastric mucosa, albeit rarely [3]. To our knowledge, endoscopic pictures and detailed pathological features of the white globe appearance in patients without gastric cancer have not been described thus far. Here, we present two patients with multiple white spots in the non-cancerous gastric mucosa, which were consistent with the known features of the white globe appearance. Here, we discuss differences in the pathological features of white globe appearance in gastric cancer versus non-cancerous stomach.

## Case presentation

### Case 1

An 82-year-old Japanese man underwent esophagogastroduodenoscopy for a routine health checkup. The patient had been taking vonoprazan, dimethicone, acotiamide, sitagliptin, candesartan, dutasteride, etizolam and zolpidem for reflux esophagitis, functional dyspepsia, diabetes, hypertension, benign prostatic hyperplasia and insomnia. Physical examination revealed no abnormalities in his abdomen. All laboratory findings were within the normal ranges, except for elevation of plasma glucose (256 mg/dL), haemoglobin A1c (7.1%) and gastrin levels (844 pg/mL, normal range: 42–200 pg/mL). He tested negative for *Helicobacter pylori* (*H. pylori*) IgG antibody.

**Figure 1.**
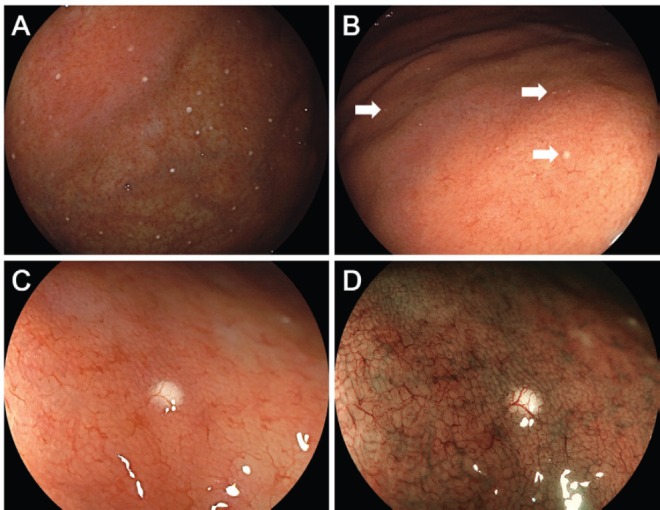
Endoscopic images of Case 1. Multiple white spots are seen in the gastric fornix (a) and body (b). Magnifying endoscopy observation (c) and blue laser imaging (d) show deposition of slightly elevated, round, white substance and microvasculature on its surface, which are consistent with reported features of white globe appearance.

**Figure 2.**
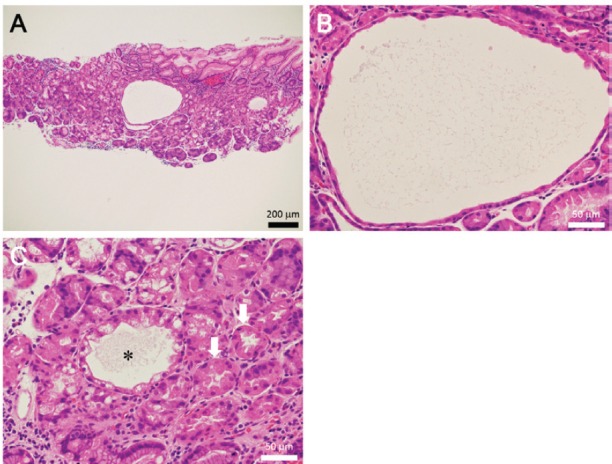
Pathology images of Case 1. Biopsied specimen reveals cystic dilatation of the gastric fundal gland that had a 400 μm diameter (a, b). Parietal cell protrusion is also noted (c, arrows) along with dilated duct (c, asterisk).

Endoscopy revealed multiple white spots in the fornix (Figure 1a) and body (Figure 1b) of the stomach. Magnifying endoscopy observation (Figure 1c) and blue laser imaging (Figure 1d) showed a slightly elevated, round, white substance. Microvasculature was also seen on its surface, suggesting deposition of the white substance within the mucosa. Atrophic gastritis was also observed during esophagogastroduodenoscopy. No inflammation was observed in the oesophageal mucosa. Biopsy from the gastric mucosa that contained white spots revealed cystic dilation of the gastric fundal gland with a 400 μm diameter (Figure 2a, b). Parietal cell protrusion was also noted (Figure 2c, arrows). Xanthoma cells were absent in the biopsied specimen.

### Case 2

A 74-year-old Japanese woman underwent esophagogastroduodenoscopy for investigation of epigastric pain and throat discomfort. She had been taking esomeprazole, rebamipide, sitagliptin, candesartan, ezetimibe, mirabegron, levocetirizine, zolpidem and lactobacillus preparation for reflux esophagitis, diabetes, hypertension, hyperlipidaemia, urticaria and insomnia. The patient had also been using flurbiprofen poultice for chronic lumbar pain. She is allergic to multiple medications, including antibiotics. Although the patient underwent eradication treatment for *H. pylori* 2 years previously, she discontinued taking the medication due to epigastric discomfort, and eradication failed. Physical examination revealed no abnormalities in her abdomen. Laboratory findings revealed elevated levels of total cholesterol (254 mg/dL), triglyceride (130 mg/dL), haemoglobin A1c (7.7%) and immunoglobulin E (598 IU/mL, normal range: 0–170 IU/mL). Gastrin levels were not measured in this patient. *H. pylori* IgG antibody was positive.

**Figure 3.**
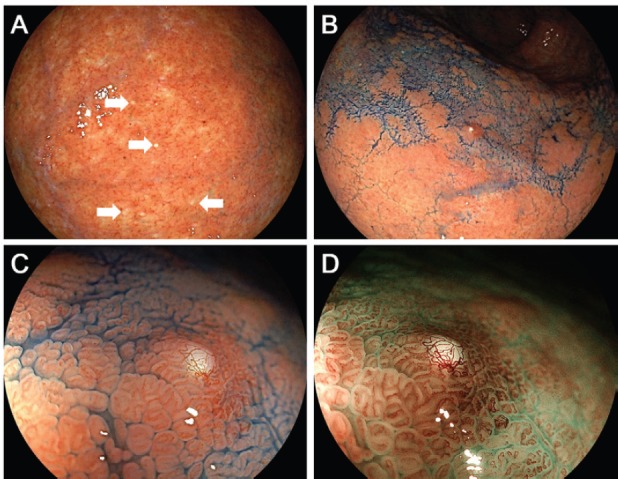
Endoscopic images of Case 2. Multiple white spots are identified in the gastric fornix (a, arrows) and body (b, post-indigo carmine spraying). Magnifying endocopy observation (c) and blue laser imaging (d) show small, round, white deposits.

**Figure 4.**
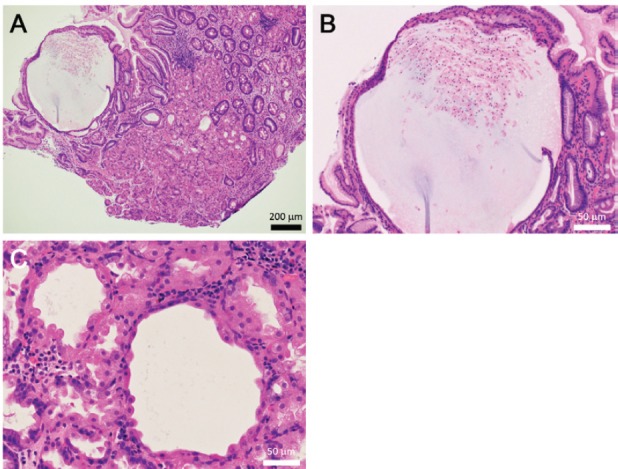
Pathology images of Case 2. The biopsied specimen shows cystic dilatation of the gastric fundal gland of approximately 600 μm diameter (a, b). Parietal cell protrusions and dilated glands forming microcysts are also seen (c).

Esophagogastroduodenoscopy revealed multiple white spots in the gastric fornix (Figure 3a, arrows), body (Figure 3b, post-indigo carmine spraying) and antrum. Magnifying endoscopy observation (Figure 3c) and blue laser imaging (Figure 3d) showed small, round, white deposits that were similar to the substances observed in case 1. Other endoscopic findings included atrophic gastritis and oesophageal hiatal hernia. Cystic dilation of the gastric fundal gland was identified in the biopsied specimen obtained from the white spots, which contained debris in the dilated duct (Figure 4a, b). The dilated duct was approximately 600 μm in diameter. In addition, parietal cell protrusions and dilated glands forming microcysts were present (Figure 4c).

## Discussion

In the presented two patients, white substance was deposited in a slightly elevated, circular fashion within the gastric mucosa, and microvasculature was observed on its surface. The white substance pathologically consisted of cystic dilation of the duct with a 400–600 μm diameter. Doyama *et al* defined the white globe appearance as a small (<1 mm) white lesion of globular shape that is found underneath the gastric epithelium and can be clearly visualised using magnifying endoscopy with narrow-band imaging [1, 4]. Microvessels overlying the white substance, reflecting the white substance existing underneath the gastric epithelium and subepithelial microvessels, have been reported to be useful for identifying the white globe appearance [3]. The endoscopic features observed in the presented two cases fit these definitions. As described earlier, to the best of our knowledge, this report is the first to present detailed endoscopic and pathologic images of the white globe appearance in cases without gastric cancer.

**Figure 5.**
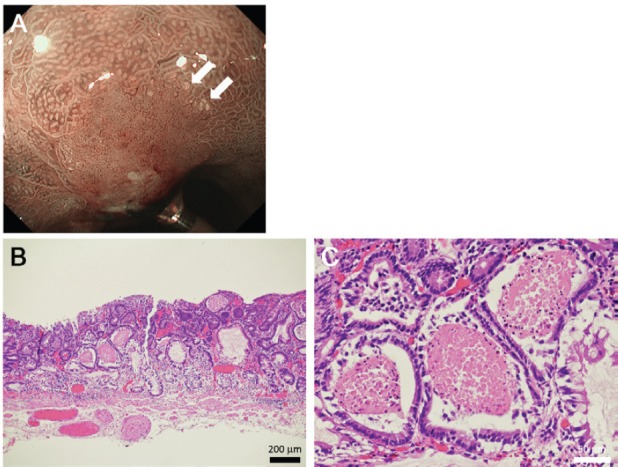
Representative images of white globe appearance observed in a patient with gastric cancer. Tiny white substances are observed in the periphery of the cancer lesion (a, arrows). Intraglandular necrotic debris is seen in the resected specimen (b, c).

As described earlier, Doyama *et al* described the white globe appearance as a novel marker to correctly diagnose early gastric cancer [1, 3]. Figure 5 shows the typical images of the white globe appearance observed in a 78-year-old Japanese man with gastric cancer.

Tiny white substances were observed in the periphery of the cancer lesion (Figure 5a, arrows). In an endoscopically resected specimen, eosinophilic materials with necrotic epithelial fragments were identified within the lumen of the dilated glands (Figure 5b, c), which are called intraglandular necrotic debris [1–3]. Meanwhile, neither eosinophilic materials nor necrotic epithelial fragments were identified in the two presented cases. We speculate that the deposited material was mucus in Case 1 and degenerated epithelial cells and mucus in Case 2. White globe features in the present two cases seemed to be more elevated and whiter than those in gastric cancer. In addition, the distribution of white globe appearance was patchy in the present two cases. Such differences of the endoscopic and pathological features between cases with and without cancer lesions probably reflect different underlying pathogeneses of the white globe appearance.

Yoshida *et al* [3] described three cases of patients without gastric cancer who showed white globe appearance in the stomach. One patient had a benign open ulcer, another had gastritis, and the other had low-grade adenoma with an ulcer scar. Because two of the three patients had a gastric ulcer, the authors speculated that ulceration is associated with the pathogenesis of the white globe appearance. However, no ulceration was detected endoscopically or pathologically in the two presented cases. In addition, the white globe appearance was distributed in wide areas (the fornix and body in Case 1 and the fornix, body and antrum in Case 2).

Several possible mechanisms can be hypothesised for the white globe appearance in the two cases. First, an acid secretion inhibitor might induce such features. The patient in Case 1 had been taking vonoprazan, a potassium-competitive acid blocker, and the patient in Case 2 had been taking esomeprazole, a proton-pump inhibitor. These acid secretion inhibitors lead to hypergastrinemia, which causes parietal cell enlargement and a serrated or tongue-like internal gland profile, that is, parietal cell protrusion [5]. Subsequently, parietal cell protrusion would obstruct outflow from the gland, finally resulting in intramucosal cyst formation of dilated glands, which is called fundic gland cysts [6]. We speculate that fluid in the fundic gland cysts was observed as the white globe appearance. The presence of parietal cell protrusion in the biopsied specimen of the presented cases supports this hypothesis. Hatano *et al* [7] recently reported black spots as a novel gastric finding, which was potentially induced by proton-pump inhibitor use. Among 64 patients, 44 (68.8%) were taking proton-pump inhibitors. Parietal cell protrusions and fundic gland cysts were pathologically identified in 26 (76.5%) and 23 (64.1%) cases, respectively. The authors speculated that the brownish pigmentation retained in the fundic gland cysts was responsible for the black spots. Although the colour was apparently different between black spots reported by Hatano *et al* and white spots observed in the presented two cases, they shared common macroscopic and microscopic features; both of them presented with small punctiform lesions in the stomach and had parietal cell protrusions and fundic gland cysts. Therefore, a common ethology may exist between the black spots and white globe appearance in the non-cancerous stomach.

The second hypothesis is that medications other than acid secretion inhibitors or underlying diseases might cause the white globe appearance. For example, both patients in Cases 1 and 2 had been taking sitagliptin, candesartan and zolpidem. Moreover, both patients had diabetes mellitus. Any of these drugs or diabetes mellitus might be involved in the pathogenesis of the white spots. Third, *H. pylori*-associated chronic gastritis might contribute to the formation of white globe appearance. The patient in Case 2 had active *H. pylori* infection and white globe appearance was observed at the border of the atrophic gastric mucosa. In Case 1, although *H. pylori* was serologically and pathologically negative, and the white globe appearance was observed in the non-atrophic gastric mucosa, the presence of atrophic gastritis meant past *H. pylori* infection. Inflammation and alteration of gastric mucosa due to *H. pylori* may promote or facilitate the formation of white spots in the stomach. Because the pathogenesis of white globe appearance has not been described thus far, further investigations are warranted.

## Conclusions

We present two cases with white globe appearance observed in the non-cancerous gastric mucosa. Pathologically, parietal cell protrusion and fundic gland cysts existed, whereas intraglandular necrotic debris was absent. The results of the present study reinforce that white globe appearance can be observed in non-cancerous stomach. Although the macroscopic features could be confusing or misleading, thorough endoscopic observation and pathological analysis of white globe appearance will aid oncologists and endoscopists in differentiating between cancer-related lesions and non-cancerous lesions.

## Conflicts of interest

The authors declare that they have no conflicts of interest regarding the publication of this paper.

## Funding

The authors did not receive any funding for this work.
